# Acetylsalicylic Acid Reduces Passive Aortic Wall Stiffness and Cardiovascular Remodelling in a Mouse Model of Advanced Atherosclerosis

**DOI:** 10.3390/ijms23010404

**Published:** 2021-12-30

**Authors:** Lynn Roth, Miche Rombouts, Dorien M. Schrijvers, Besa Emini Veseli, Wim Martinet, Guido R. Y. De Meyer

**Affiliations:** Laboratory of Physiopharmacology, University of Antwerp, 2610 Antwerp, Belgium; micherombouts@gmail.com (M.R.); dorien.schrijvers@histogenex.com (D.M.S.); Besa.Emini@uantwerpen.be (B.E.V.); wim.martinet@uantwerpen.be (W.M.); guido.demeyer@uantwerpen.be (G.R.Y.D.M.)

**Keywords:** arterial stiffness, neutrophil–lymphocyte ratio, collagen, SMAD, elastin, aspirin

## Abstract

Acetylsalicylic acid (ASA) is widely used in secondary prevention of cardiovascular (CV) disease, mainly because of its antithrombotic effects. Here, we investigated whether ASA can prevent the progression of vessel wall remodelling, atherosclerosis, and CV complications in apolipoprotein E deficient (*ApoE^−/−^*) mice, a model of stable atherosclerosis, and in *ApoE^−/−^* mice with a mutation in the fibrillin-1 gene (*Fbn1^C1039G+/−^*), which is a model of elastic fibre fragmentation, accompanied by exacerbated unstable atherosclerosis. Female *ApoE^−/−^* and *ApoE^−/−^Fbn1^C1039G+/−^* mice were fed a Western diet (WD). At 10 weeks of WD, the mice were randomly divided into four groups, receiving either ASA 5 mg/kg/day in the drinking water (*ApoE^−/−^* (*n* = 14), *ApoE^−/−^Fbn1^C1039G+/−^* (*n* = 19)) or plain drinking water (*ApoE^−/−^* (*n* = 15), *ApoE^−/−^Fbn1^C1039G+/−^* (*n* = 21)) for 15 weeks. *ApoE^−/−^Fbn1^C1039G+/−^* mice showed an increased neutrophil–lymphocyte ratio (NLR) compared to *ApoE^−/−^* mice, and this effect was normalised by ASA. In the proximal ascending aorta wall, ASA-treated *ApoE^−/−^Fbn1^C1039G+/−^* mice showed less p-SMAD2/3 positive nuclei, a lower collagen percentage and an increased elastin/collagen ratio, consistent with the values measured in *ApoE^−/−^* mice. ASA did not affect plaque progression, incidence of myocardial infarction and survival of *ApoE^−/−^Fbn1^C1039G+/−^* mice, but systolic blood pressure, cardiac fibrosis and hypertrophy were reduced. In conclusion, ASA normalises the NLR, passive wall stiffness and cardiac remodelling in *ApoE^−/−^Fbn1^C1039G+/−^* mice to levels observed in *ApoE^−/−^* mice, indicating additional therapeutic benefits of ASA beyond its classical use.

## 1. Introduction

Age-related diseases such as cardiovascular disease (CVD), have a substantial impact on our quality of life. Vascular ageing is characterised by structural and functional changes in the wall of large arteries leading to arterial stiffness, thereby promoting atherosclerotic disease progression [1]. Atherosclerosis is a progressive inflammatory disease of the large and medium-sized arteries, characterised by the formation of plaques in the vessel wall. During the development of the disease, the stability of the atherosclerotic plaque plays a major role. Features of plaque instability include a large necrotic core, a high infiltration of inflammatory macrophages and a thin fibrous cap, composed of few smooth muscle cells (SMCs) and collagen fibres. When a plaque develops such an unstable phenotype, it may easily rupture, followed by thrombosis and clinical complications such as myocardial infarction (MI) and stroke [2,3,4]. Despite the significant therapeutic advances in cardiology over the past decades, atherosclerotic plaque rupture remains a leading cause of acute cardiovascular death. Delaying the process of vascular ageing and arterial stiffening might reduce the progression of atherosclerosis, thereby reducing the societal and economic burden of CVD.

Extracellular matrix remodelling plays an essential role in vascular ageing and arterial stiffness. With age, mechanical fracture of elastin and subsequent proteolysis will become more abundant, hence reducing the elasticity of the vessel wall. Furthermore, collagen synthesis will be increased, mainly because of enhanced TGF-β signalling, which will further promote arterial stiffening. In the end, this will lead to increased systolic blood pressure (BP), cardiac remodelling, and atherogenesis [5]. Another important characteristic of ageing, is chronic low-grade inflammation, termed ‘inflammaging’, which also contributes to arterial stiffness. The neutrophil–lymphocyte ratio (NLR) is a biomarker of chronic inflammation that increases with age [6,7,8]. Importantly, NLR is associated with arterial stiffness and increased morbidity and mortality in CVD patients [6,9,10,11,12,13]. Measuring NLR could therefore provide a good indication of the severity of age-related vascular stiffness and the associated progression of CVD. Overall, targeting vascular remodelling and inflammation could be a valuable approach to attenuate the progression of arterial stiffness and atherosclerosis. Current therapeutic strategies to prevent or treat vascular ageing are lifestyle interventions, such as physical activity, caloric restriction, and maintaining a healthy diet. Furthermore, pharmacological interventions with a proven effect on arterial stiffness include anti-hypertensive agents, statins, mTOR inhibitors (e.g., rapamycin), and AMPK activators (e.g., metformin), whereas PCSK9 monoclonal antibodies are promising [14,15]. Nevertheless, the need for novel therapeutic strategies remains high, due to the current ageing population and consequent higher burden of age-related diseases. In this regard, a good approach would be to evaluate known drugs, used in CVD prevention, for their potential to inhibit vascular remodelling and the development of arterial stiffness.

Acetylsalicylic acid (ASA) has been used for many years in the treatment and prevention of CVD [16,17,18]. It is an irreversible inhibitor of the enzyme cyclooxygenase and belongs to the class of the nonsteroidal anti-inflammatory drugs. Low doses of ASA (80–100 mg) are sufficient to block cyclooxygenase-1 and thus possess the ability to inhibit platelet aggregation [16]. In primary prevention, ASA is not recommended due to an increased bleeding risk (class III ESC guidelines), but in secondary prevention after MI or stroke the risk reduction is very significant even in patients with stable cardiovascular disease (class I ESC guidelines) [19,20,21,22].

The goal of the present study was to test the effect of low-dose ASA on the progression of vessel wall remodelling, atherosclerosis, and cardiovascular complications in apolipoprotein E deficient (*ApoE^−/−^*) mice, the classical model to study atherosclerosis, and *ApoE^−/−^* mice with a heterozygous mutation in the fibrillin-1 gene (*Fbn1^C1039G+/−^*). Mutations in the *Fbn1* gene result in an impaired microfibrillar assembly and deposition, followed by fragmentation of elastic fibres and increased collagen deposition. This loss of structural integrity of the vessel wall leads to progressive dilatation and arterial stiffening, resembling vascular ageing [23,24,25,26,27]. In addition, the *ApoE^−/−^Fbn1^C1039G+/−^* mouse model shows accelerated atherosclerotic plaque progression, spontaneous plaque ruptures, MI, and sudden death [28,29]. It is therefore an excellent model to study pharmacological interventions targeting vascular ageing and CVD, because it combines two important risk factors, hypercholesterolemia (*ApoE^−/−^*) and arterial stiffness (*Fbn1^C1039G+/−^*).

## 2. Results

**Figure 1:**
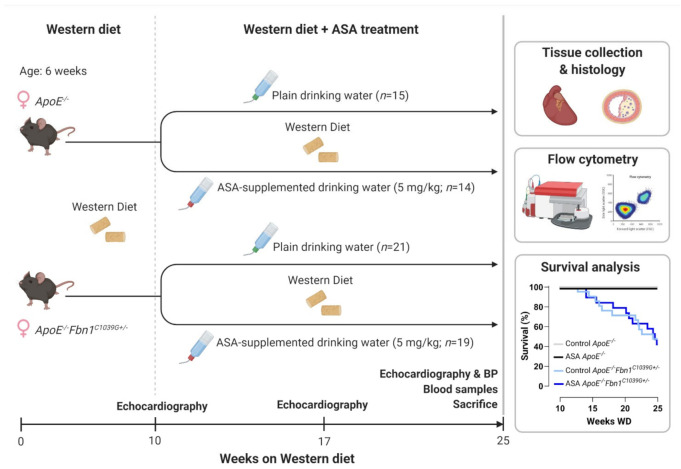
Experimental outline and Kaplan–Meier survival curve. BP = blood pressure; WD = Western diet. Created with BioRender.com (accessed on 30 December 2021).

A general overview of the study outline, timing and treatment groups is visualised in Figure 1. Mortality, caused by sudden death of the mice during the timeframe of the treatment protocol, was significantly higher in *ApoE^−/−^Fbn1^C1039G+/−^* mice compared to *ApoE^−/−^* mice, but was not affected by ASA treatment (Table 1, Figure 1). Importantly, no major bleeding complications were detected in ASA-treated mice. Total plasma cholesterol levels were not different and body weight was reduced in *ApoE^−/−^Fbn1^C1039G+/−^* mice compared to *ApoE^−/−^* controls, without a significant treatment effect (Table 1). Heart, lung, and spleen weight were all normalised over body weight and increased in *ApoE^−/−^Fbn1^C1039G+/−^* mice. Furthermore, heart and spleen weight in *ApoE^−/−^Fbn1^C1039G+/−^* mice were reduced as a result of ASA treatment (Table 1).

### 2.1. ASA Treatment Normalises Neutrophil–Lymphocyte Ratio in ApoE^−/−^Fbn1^C1039G+/−^ Mice

**Figure 2:**
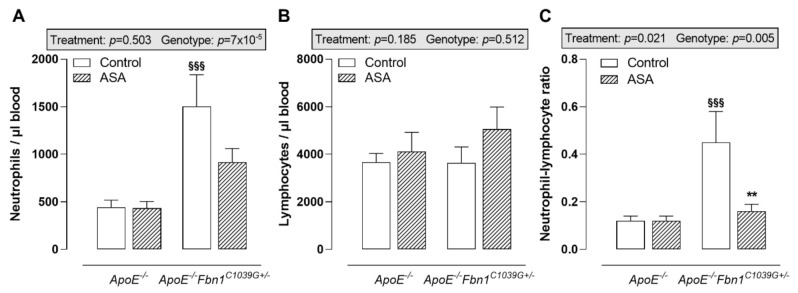
Blood neutrophils and lymphocytes. (**A**) Circulating neutrophils were higher in control *ApoE^−/−^Fbn1^C1039G+/−^* mice compared to *ApoE^−/−^* mice and were not significantly affected by ASA treatment. (**B**) Absolute numbers of circulating lymphocytes remained equal between all groups of mice. (**C**) As a result of ASA treatment, the neutrophil–lymphocyte ratio was normalised in *ApoE^−/−^Fbn1^C1039G+/−^* mice. 2-way ANOVA: ^§§§^ *p* < 0.001 vs. *ApoE^−/−^* control and ** *p* < 0.01 vs. *ApoE^−/−^Fbn1^C1039G+/−^* control.

The analysis of circulating immune cells clearly showed higher neutrophil levels in the blood of control *ApoE^−/−^Fbn1^C1039G+/−^* mice compared to *ApoE^−/−^* mice, while lymphocyte levels remained equal (Figure 2A,B). In response to ASA treatment, a trend towards lower circulating neutrophils was observed in *ApoE^−/−^Fbn1^C1039G+/−^* mice (Figure 2A). After calculating the neutrophil–lymphocyte ratio, a significant increase could be observed in control *ApoE^−/−^Fbn1^C1039G+/−^* mice. This increase was abolished in the ASA-treated group (Figure 2C). In *ApoE^−/−^Fbn1^C1039G+/−^* mice, the percentage of circulating Ly-6C^hi^ monocytes was higher and ASA treatment was not able to normalise this pattern (Table 2). The levels of dendritic cells, Ly-6C^lo^ monocytes, T cells, B cells, NK, and NKT cells were not affected by genotype or treatment (Table 2).

### 2.2. Atherosclerotic Plaque Progression and the Occurrence of Myocardial Infarctions Are Not Affected by ASA

*ApoE^−/−^Fbn1^C1039G+/−^* mice showed a marked increase in plaque size, stenosis, and vessel dilatation (analysed by measuring internal elastic lamina length) in the proximal ascending aorta compared to *ApoE^−/−^* mice. ASA treatment was not able to reduce these disease parameters (Table 3, Figure S1). Furthermore, the stability of the plaques remained equal between control and ASA-treated mice, since no changes in necrotic core size, percentage of smooth muscle cells, fibrous cap thickness, total collagen content, percentage of glycosaminoglycans, and the presence of leukocytes and macrophages were observed (Table 3, Figure S1). In addition, we could not observe a difference in p-SMAD2/3 positive nuclei in both control and ASA-treated *ApoE^−/−^* and *ApoE^−/−^Fbn1^C1039G+/−^* mice (Table 3, Figure S1).

In the common carotid artery, plaque formation index was higher in *ApoE^−/−^Fbn1^C1039G+/−^* mice compared to *ApoE^−/−^* mice, but it was not affected by ASA treatment (Table 3). Neither the genotype nor treatment affected the presence of intraplaque microvessels and bleeding (Table 3).

The percentage of mice showing plaques in the coronary arteries was not different between genotypes and treatment groups (Table 3). Furthermore, plaque size and stenosis were equal (Table 3). The occurrence of MI was higher in *ApoE^−/−^Fbn1^C1039G+/−^* mice and was not altered by ASA treatment (Table 3).

### 2.3. Passive Wall Stiffness Is Reduced in ASA-Treated ApoE^−/−^Fbn1^C1039G+/−^ Mice

**Figure 3:**
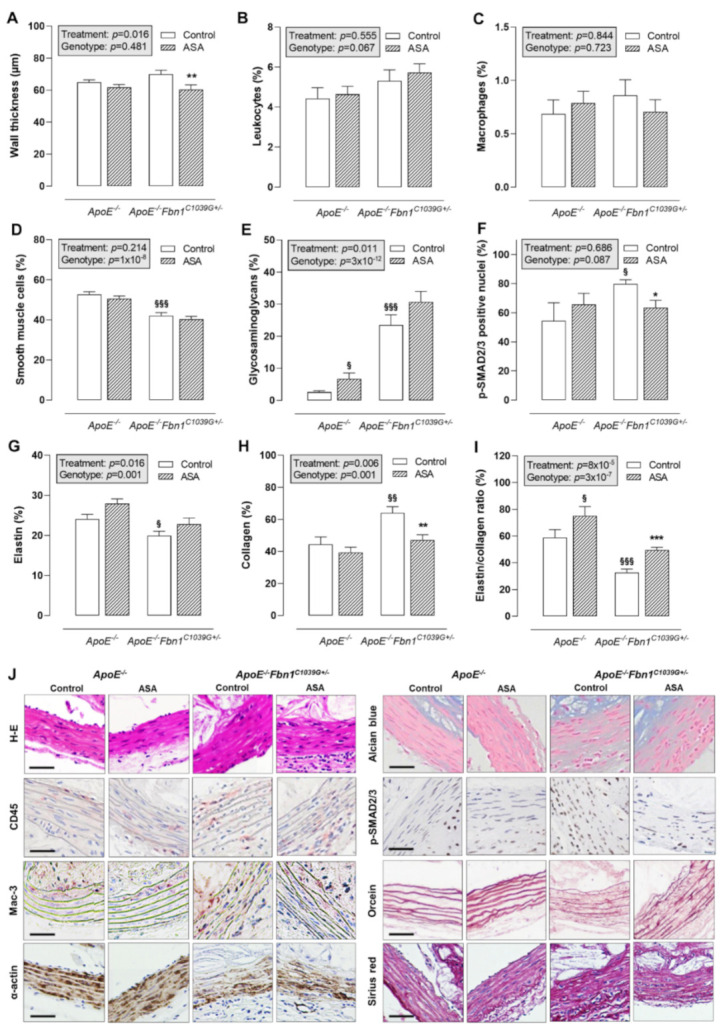
Thickness and composition of the proximal ascending aorta wall. (**A**) H-E staining was used to evaluate the thickness of the vessel wall, which was reduced as a result of ASA treatment in *ApoE^−/−^Fbn1^C1039G+/−^* mice. (**B**,**C**) The percentage of leukocytes (CD45 positive cells) and macrophages (Mac-3 positive area) in the vessel wall did not change. (**D**) The α-actin positive area (smooth muscle cells) was significantly lower in *ApoE^−/−^Fbn1^C1039G+/−^* mice and was not affected by ASA treatment. (**E**) Alcian blue staining indicated an increase in glycosaminoglycans in *ApoE^−/−^Fbn1^C1039G+/−^* mice as compared to *ApoE^−/−^* mice. In addition, ASA treatment resulted in a higher percentage of glycosaminoglycans in *ApoE^−/−^* mice. (**F**) The percentage of p-SMAD2/3 positive nuclei were higher in *ApoE^−/−^Fbn1^C1039G+/−^* control mice as compared to *ApoE^−/−^* mice. ASA treatment was able to reduce p-SMAD2/3 positivity in *ApoE^−/−^Fbn1^C1039G+/−^* mice. (**G**) Orcein staining clearly showed the fragmentation of elastic fibres and a lower elastin content in *ApoE^−/−^Fbn1^C1039G+/−^* mice, which was not significantly affected by ASA treatment. (**H**) Collagen content of the vessel wall (Sirius red positive area) was higher in control *ApoE^−/−^Fbn1^C1039G+/−^* mice and normalised by ASA treatment. (**I**) The elastin/collagen ratio was reduced in *ApoE^−/−^Fbn1^C1039G+/−^* mice and ASA treatment led to an increase in both models. (**J**) Representative images of the different stainings. Scale bar = 50 µm. 2-way ANOVA: ^§^ *p* < 0.05, ^§§^ *p* < 0.01 ^§§§^ *p* < 0.001 vs. control *ApoE^−/−^* and * *p* < 0.05, ** *p* < 0.01, *** *p* < 0.01 vs. control *ApoE^−/−^Fbn1^C1039G+/−^*.

Histological analysis of the proximal ascending aorta wall showed that ASA treatment was able to reduce wall thickness in *ApoE^−/−^Fbn1^C1039G+/−^* mice, but not in *ApoE^−/−^* mice (Figure 3A,J). The total leukocyte and macrophage content of the vessel wall was rather low and equal in all groups (Figure 3B,C,J). The percentage of α-actin positive smooth muscle cells was significantly lower in *ApoE^−/−^Fbn1^C1039G+/−^* mice and was not affected by ASA (Figure 3D,J). The presence of glycosaminoglycans in the vessel wall of *ApoE^−/−^Fbn1^C1039G+/−^* mice was increased as compared to *ApoE^−/−^* mice. In addition, ASA treatment resulted in a higher percentage of glycosaminoglycans in *ApoE^−/−^* mice, but not in *ApoE^−/−^Fbn1^C1039G+/−^* mice (Figure 3E,J). The percentage of p-SMAD2/3 positive nuclei was higher in *ApoE^−/−^Fbn1^C1039G+/−^* control mice as compared to *ApoE^−/−^* mice. In addition, ASA treatment was able to reduce p-SMAD2/3 positivity in *ApoE^−/−^Fbn1^C1039G+/−^* mice to the levels observed in *ApoE^−/−^* mice (Figure 3F,J). Elastin fragmentation was clearly present in *ApoE^−/−^Fbn1^C1039G+/−^* mice and the number of elastin breaks was significantly higher as compared to *ApoE^−/−^* mice in both control (*ApoE^−/−^*: 17 [11–23]; *ApoE^−/−^Fbn1^C1039G+/−^*: 114 [54–176]; *p* = 0.000095) and ASA-treated groups (*ApoE^−/−^*: 17 [12–22]; *ApoE^−/−^Fbn1^C1039G+/−^*: 118 [31–194]; *p* = 0.000027; Kruskal–Wallis test with Bonferroni correction for multiple comparisons). However, ASA treatment did not significantly affect the number of elastin breaks in *ApoE^−/−^* mice (*p* = 0.905) and in *ApoE^−/−^Fbn1^C1039G+/−^* mice (*p* = 0.977; Kruskal–Wallis test with Bonferroni correction for multiple comparisons). The percentage of elastin positive area was decreased in *ApoE^−/−^Fbn1^C1039G+/−^* mice as compared to *ApoE^−/−^* control mice and in accordance with the number of elastin breaks, ASA treatment did not affect elastin content of the vessel wall in both models (Figure 3G,J). A higher collagen fibre content was observed in *ApoE^−/−^Fbn1^C1039G+/−^* mice compared to *ApoE^−/−^* mice, which was normalised by treatment with ASA (Figure 3H,J). After calculating the elastin/collagen ratio, a distinct decrease was detected in *ApoE^−/−^Fbn1^C1039G+/−^* mice. Interestingly, ASA treatment led to higher elastin/collagen ratios in both mouse models (Figure 3I,J).

### 2.4. ASA Treatment Reduces Systolic Blood Pressure and Cardiac Remodelling in ApoE^−/−^Fbn1^C1039G+/−^ Mice

**Figure 4:**
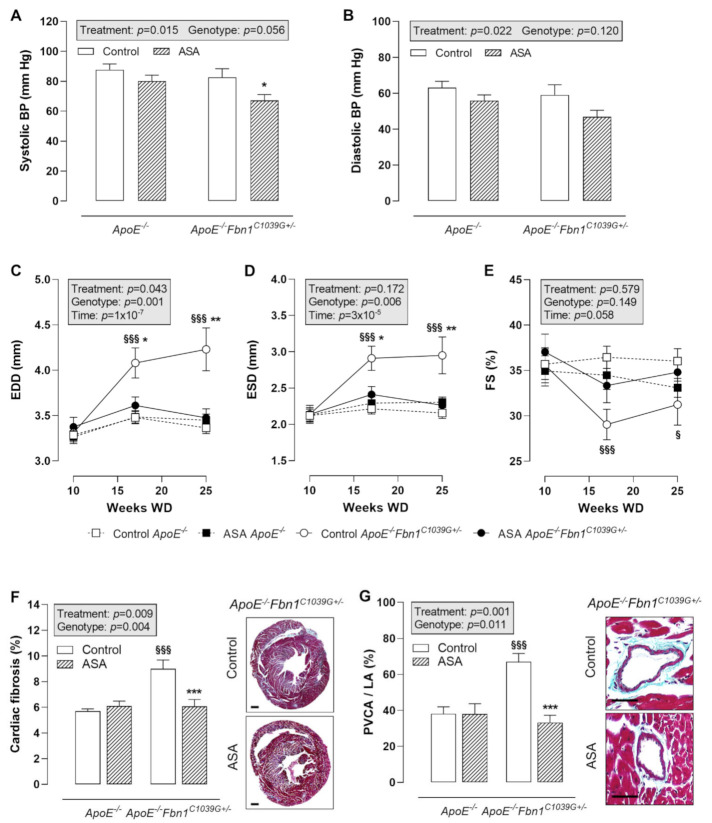
Blood pressure and cardiac remodelling. (**A**,**B**) Systolic blood pressure (BP) was lower after ASA treatment in *ApoE^−/−^Fbn1^C1039G+/−^* mice. Diastolic BP was not significantly reduced. 2-way ANOVA: * *p* < 0.05 vs. control *ApoE^−/−^Fbn1^C1039G+/−^*. (**C**–**E**) End-diastolic diameter (EDD) and end-systolic diameter (ESD) were normalised in ASA-treated *ApoE^−/−^Fbn1^C1039G+/−^* mice. A trend towards higher fractional shortening (FS) was observed in *ApoE^−/−^Fbn1^C1039G+/−^* mice treated with ASA. 3-way mixed ANOVA: ^§^ *p* < 0.05, ^§§§^ *p* < 0.001 vs. control *ApoE^−/−^* and * *p* < 0.05, ** *p* < 0.01 vs. ASA *ApoE^−/−^Fbn1^C1039G+/−^* (**F**,**G**) Total cardiac fibrosis (Trichrome Masson staining; scale bar = 500 µm) was increased in control *ApoE^−/−^Fbn1^C1039G+/−^* mice and normalised after ASA treatment. Trichrome Masson staining of the coronary arteries (scale bar = 50 µm) revealed a significant reduction of perivascular fibrosis (perivascular collagen area divided by luminal area = PVCA/LA) after ASA treatment in *ApoE^−/−^Fbn1^C1039G+/−^* mice. 2-way ANOVA: ^§§§^ *p* < 0.001 vs. control *ApoE^−/−^* and *** *p* < 0.001 vs. control *ApoE^−/−^Fbn1^C1039G+/−^*.

Blood pressure (BP) measurements at 25 weeks of WD revealed a significantly lower systolic BP in ASA-treated *ApoE^−/−^Fbn1^C1039G+/−^* mice compared to control *ApoE^−/−^Fbn1^C1039G+/−^* mice (Figure 4A). In *ApoE^−/−^* mice, the same trend could be observed. Diastolic BP was not significantly changed, although a decreasing trend was present as a result of ASA treatment in both mouse models (Figure 4B).

Echocardiography of the heart showed a clear increase in end-diastolic diameter (EDD) and end-systolic diameter (ESD) in control *ApoE^−/−^Fbn1^C1039G+/−^* mice at week 17 and 25 of WD, which was normalised to the level of *ApoE^−/−^* mice after ASA treatment (Figure 4C,D). Fractional shortening (FS) was significantly decreased at week 17 and 25 of WD in *ApoE^−/−^Fbn1^C1039G+/−^* vs. *ApoE^−/−^* mice. A trend towards higher values was observed after ASA treatment, equal to FS measured in *ApoE^−/−^* mice (Figure 4E). Total cardiac fibrosis (Figure 4F) and coronary perivascular fibrosis (Figure 4G) clearly normalised in *ApoE^−/−^Fbn1^C1039G+/−^* mice after treatment with ASA.

## 3. Discussion

In the present study, we investigated whether ASA therapy has an additional benefit in CVD prevention by reducing vessel wall remodelling and atheroprogression. The effects were compared between *ApoE^−/−^* and *ApoE^−/−^Fbn1^C1039G+/−^* mice in order to analyse two different stages of disease severity. *ApoE^−/−^* mice are a classical model to study atherosclerosis. They develop atherosclerotic plaques of different stages, but have the disadvantage that plaque rupture and complications such as MI and sudden death are rare, which are the most important aspects of human atherosclerosis [30]. Therefore, we included the *ApoE^−/−^Fbn1^C1039G+/−^* mouse model as well. Mutations in the *Fbn1* gene lead to fragmentation of elastic fibres [31]. This results in increased arterial stiffening and progressive aortic dilatation, mimicking vascular ageing [24,25,29]. Moreover, in *ApoE^−/−^* mice, this mutation leads to the development of highly unstable plaques, resulting in spontaneous plaque rupture with end-points including MI and sudden death [28,29]. Thus, *ApoE^−/−^Fbn1^C1039G+/−^* mice show many features of human end-stage atherosclerosis and are therefore a good model for evaluating the therapeutic effects of drugs on age-related vessel wall remodelling, established atherosclerosis, and its complications.

Clear signs of chronic inflammation were observed in *ApoE^−/−^Fbn1^C1039G+/−^* mice, since the number of circulating Ly-6C^hi^ monocytes, neutrophils and the NLR were significantly higher compared to *ApoE^−/−^* mice. The NLR is an inflammatory biomarker that independently predicts cardiovascular risk and mortality in humans [13]. It is important to note that in humans a normal NLR is between 1 and 3, while in mice these values are lower, in the range of 0.1–0.3 [32]. It was striking that ASA treatment was able to normalise this parameter. This finding is in accordance with published data, describing a lower NLR in coronary artery disease patients receiving ASA therapy [33]. Many risk factors of CVD promote neutrophilia. For instance, metabolic changes, such as hypercholesterolemia and hyperglycaemia, stress, ageing, and sleep disorders accelerate myelopoiesis. The resulting increase in circulating neutrophils plays an important role in atheroprogression by secreting reactive oxygen species, proteases, and myeloperoxidase, resulting in endothelial activation and monocyte recruitment [34,35,36]. Although we could observe a decrease in NLR in *ApoE^−/−^Fbn1^C1039G+/−^* mice after ASA treatment, we could not observe a similar anti-inflammatory trend in atherosclerotic plaques of these mice. In addition, no effect on plaque size and stability was observed. We initiated ASA treatment at 10 weeks of Western diet, a time point at which advanced plaques are present in *ApoE^−/−^Fbn1^C1039G+/−^* mice, as observed by Van Herck et al. [29]. It is possible that our treatment protocol was not sufficient to modulate plaque inflammation, due to the excessive presence of inflammatory cytokines and matrix degrading enzymes in plaques of *ApoE^−/−^Fbn1^C1039G+/−^* mice at the start of ASA administration [28].

The vessel wall of *ApoE^−/−^Fbn1^C1039G+/−^* mice showed clear signs of remodelling. We observed a significantly lower elastin/collagen ratio compared to *ApoE^−/−^* mice, which is an important indicator of passive wall stiffness. This was mainly due to a marked increase in total collagen content. In addition, the presence of glycosaminoglycans was dramatically increased. TGF-β is considered an important mediator in this remodelling process. Microfibrils interact with TGF-β-binding proteins. Thus, a mutation in the *Fbn1* gene results in increased TGF-β levels and activity, due to disturbed sequestration of this cytokine. Ultimately, this will lead to a higher collagen and glycosaminoglycan production in the vessel wall [37,38,39]. We observed an increase in p-SMAD2/3 positive nuclei in the vessel wall of *ApoE^−/−^Fbn1^C1039G+/−^* mice, clearly demonstrating enhanced TGF-β signalling. Interestingly, ASA treatment resulted in a lower percentage of p-SMAD2/3 positive nuclei in the proximal ascending aorta wall of *ApoE^−/−^Fbn1^C1039G+/−^* mice. In contrast, we also observed a slight increase in glycosaminoglycans. Whether this will be harmful or affect vascular function after chronic treatment, remains to be investigated. Apart from this finding, we observed a clear reduction in passive wall stiffness, as seen by a lower collagen content and a subsequent increase in the elastin/collagen ratio. It has been described that ASA treatment is able to reduce aortic stiffness in humans [40,41]. Thus, based upon our findings, it is plausible that the decrease in SMAD signalling plays a major role in this beneficial property. This is also in accordance with a study by Franken et al., that investigated the effect of anti-inflammatory therapies on aortic dilatation and remodelling in *Fbn1^C1039G+/−^* mice and found that reducing inflammation was not sufficient to improve disease progression. Only the use of losartan, which is an Angiotensin II type 1 receptor blocker, was able to induce a therapeutic benefit by reducing both inflammation and SMAD signalling [42]. It has been demonstrated that ASA is able to reduce the transcription of the Angiotensin II type 1 receptor in cardiac fibroblasts. This resulted in a reduction in collagen synthesis, which was comparable to the effect of losartan [43]. Another publication described that ASA was able to reduce SMAD signalling in the kidney of diabetic rats, which further supports our data [44].

Subsequent to the effects on vessel wall remodelling, we investigated related blood pressure and cardiac parameters. Arterial stiffness is known to be both a cause and consequence of hypertension [45]. Although we could not observe a higher systolic BP in *ApoE^−/−^Fbn1^C1039G+/−^* mice compared to *ApoE^−/−^* mice, we did see a decrease after treatment with ASA, which may be the result of the lower passive wall stiffness. Due to the positive feedback loop between hypertension and arterial stiffness, the reduction in systolic BP may also have contributed to the observed decrease in passive stiffness [45]. In addition, a marked reduction in left ventricular dilatation, cardiac fibrosis and heart weight was observed in ASA-treated *ApoE^−/−^Fbn1^C1039G+/−^* mice. This finding is not unexpected due to the lower systolic BP and reduced stiffness of the proximal ascending aorta wall, thereby minimizing hemodynamic overload. It is also plausible that, in accordance to the vessel wall, a reduction in SMAD signalling contributed to the diminished cardiac fibrosis [43].

Besides very clear signs of left ventricular dilatation and an increase in heart weight in *ApoE^−/−^Fbn1^C1039G+/−^* mice, also spleen and lung weight were significantly higher as compared to *ApoE^−/−^* mice. It is well known that left ventricular dysfunction results in pulmonary congestion and increased lung weight [46]. In addition, it has been previously described that mice with ischemic heart failure consistently showed splenomegaly and splenic remodelling. This resulted in a mobilization of splenic proinflammatory monocytes to the heart, thereby enhancing disease progression [47,48]. Interestingly, ASA treatment was able to reduce both cardiac remodelling and spleen weight in *ApoE^−/−^Fbn1^C1039G+/−^* mice, thus it might diminish the detrimental effects related to the cardiosplenic axis.

Our finding that ASA failed to reduce MI and improve survival contrasts with the results in humans, where ASA has been used for many years in the prevention of cardiovascular disease [16,17,18]. This discrepancy may be attributed to the fibrinolytic system of mice. In these animals, plasma levels of plasminogen activator inhibitor and thrombin activatable fibrinolysis inhibitor are lower than in humans, shifting the fibrinolytic balance towards enhanced lysis of thrombi [49]. Thus, if plaque rupture occurs in *ApoE^−/−^Fbn1^C1039G+/−^* mice, this will not always give rise to occlusive thrombi that provoke MI or stroke. The observed complications are more likely the result of stenotic occlusion of arteries. Another possibility to explain the difference could be the duration of the study and/or the duration of the ASA treatment.

In conclusion, the present study showed that ASA treatment significantly improves passive wall stiffness, systolic BP and cardiac remodelling in a mouse model of elastic fibre fragmentation and advanced atherosclerosis. Furthermore, a marked reduction of NLR was observed, which is an important biomarker of chronic inflammation and is associated with adverse outcome in CVD patients. These data point towards additional benefits of ASA treatment in the prevention of age-related arterial stiffness. Furthermore, ASA might also have the potential to reduce vessel wall remodelling observed in Marfan syndrome. In order to determine the clinical significance of our findings, larger trials and more in-depth research is required.

## 4. Materials and Methods

### 4.1. Mice

Female *ApoE^−/−^* and *ApoE^−/−^Fbn1^C1039G+/−^* mice were fed a Western diet (WD, 4021.90, AB Diets, Woerden, The Netherlands) starting at the age of six weeks. The animals were group-housed (5–7 per cage) in standard mouse cages in the animal facility of the University of Antwerp, in a 12-h light/dark cycle. Cages were stored at constant room temperature (20–24 °C) and humidity (45%). Food and water were supplied ad libitum. Cases of sudden death were documented. At the end of the experiment (25 weeks of WD), blood samples were obtained from the retro-orbital plexus of anesthetised mice (ketamine 100 mg/kg, xylazine 10 mg/kg, i.p.). Subsequently, the mice were sacrificed with sodium pentobarbital (250 mg/kg, i.p.). The spleen, lung, and heart weights were determined. Analysis of total plasma cholesterol was performed via a commercially available kit (Randox, Crumlin, UK). The animal procedures were performed conform to the guidelines from Directive 2010/63/EU of the European Parliament on the protection of animals used for scientific purposes and all experiments were approved by the ethics committee of the University of Antwerp.

### 4.2. Acetylsalicylic Acid Treatment

At 10 weeks of WD, *ApoE^−/−^* and *ApoE^−/−^Fbn1^C1039G+/−^* mice were randomly divided into four groups, receiving either ASA (Aspegic^®^, 9 mg lysine acetylsalicylate is equivalent to 5 mg ASA, 14 *ApoE^−/−^* mice and 19 *ApoE^−/−^Fbn1^C1039G+/−^* mice) at a dose of 5 mg/kg/day added to the drinking water or plain drinking water (control, 15 *ApoE^−/−^* mice and 21 *ApoE^−/−^Fbn1^C1039G+/−^* mice) for 15 weeks. For stability reasons, the ASA drinking water was changed every day.

### 4.3. Echocardiography

Transthoracic echocardiograms were performed at the start of the ASA treatment (10 weeks of WD), at 17 weeks of WD and at the end of the experiment. The procedure was performed on anesthetised mice (sevoflurane; 8% for induction and 4.5% for maintenance, SevoFlo^®^, Penlon vaporiser) using a Toshiba diagnostic ultrasound system (SSA-700A), equipped with a 15-MHz transducer. EDD and ESD were measured and FS was calculated as follows: (EDD − ESD)/EDD × 100.

### 4.4. Blood Pressure Measurements

Peripheral BP was measured at 25 weeks of WD in anesthetised mice (ketamine 100 mg/kg, xylazine 10 mg/kg, i.p.) via a tail cuff (Coda^TM^ High throughput, Kent Scientific, Torrington, CT, USA). Mice were placed on a warming platform (37 °C) and 10 min of acclimatization was allowed before measurements were initiated. The mean of at least 10 measurements per mouse was used.

### 4.5. Flow Cytometry

EDTA-treated blood (500 µL) was lysed using red blood cell lysing buffer Hybri-Max (Sigma-Aldrich, St. Louis, MO, USA). Subsequently, the remaining leukocytes were labelled with the following antibodies (BioLegend, San Diego, CA, USA): anti-CD11c (N418), anti-I-Ab (KH74), anti-CD11b (M1/70), anti-CD3ε (145-2C11), anti-CD19 (6D5), anti-NK1.1 (PK136), anti-Ly-6C (HK1.4), and anti-Gr-1 (RB6-8C5). Labelling occurred in the dark at 4 °C in FACS buffer (PBS + 0.1% BSA (Sigma Aldrich, St. Louis, MO, USA) + 0.05% NaN3 (Merck, Kenilworth, NJ, USA)) containing CD16/32 Fc-receptor blocker (BioLegend, San Diego, CA, USA). Next, cells were analysed on a BD Accuri C6 cytometer equipped with a blue and red laser (Becton Dickinson, Erembodegem, Belgium). Dead cells were excluded based on forward scatter, side scatter and positive staining for propidium iodide (Invitrogen, Waltham, MA, USA). Data analysis was performed with FCS Express 4 (De Novo Software, Glendale, CA, USA).

### 4.6. Histology

After sudden death or sacrifice of the mice, the proximal ascending aorta and the heart were collected. Tissues were fixed in 4% formaldehyde for 24 h, dehydrated overnight in 60% isopropanol and embedded in paraffin. Serial cross sections (5 µm) of the proximal ascending aorta and heart were prepared for histological analyses. Atherosclerotic plaque size, stenosis (plaque size/total vessel area), necrotic core (defined as acellular areas with a threshold of 3000 µm^2^), internal elastic lamina length and wall thickness of the proximal ascending aorta were analysed using haematoxylin-eosin (H-E) stained sections. Immunohistochemical staining of the proximal ascending aorta with primary antibodies against CD45 (ab10558, Abcam, Cambridge, UK) and Mac-3 (553322, Pharmingen, San Diego, CA, USA) was used to determine the percentage of leukocytes and macrophages, respectively. Collagen content was measured in Sirius red stained sections. An α-SMC actin (F3777, Sigma, St. Louis, MO, USA) immunohistochemical staining was used to define the total vascular smooth muscle cell content in the plaque and vessel wall. Fibrous cap thickness was determined as the median value of 10 measurements of the luminal α-SMC actin positive layer of the atherosclerotic plaque. Vessel wall elastin content was measured using orcein stained sections and alcian blue staining was performed to visualise glycosaminoglycans. p-SMAD2/3 (3101, Cell Signalling, Danvers, MA, USA) immunohistochemical staining was used to determine the percentage of p-SMAD2/3 positive nuclei over the total number of nuclei in the plaque and proximal ascending aorta wall. The occurrence of MI, coronary plaques, total cardiac fibrosis, and perivascular fibrosis, measured as the mean perivascular collagen area divided by the luminal area (PVCA/LA) of 10 coronary arteries per mouse, was analysed on Masson’s trichrome stained sections of the heart. If plaques were present in the coronary arteries, plaque size and percentage stenosis were measured using Masson’s trichrome stained sections. Longitudinal sections of the carotid artery were used to analyse plaque formation index, the occurrence of intraplaque microvessels and bleeding. The plaque formation index was determined on H-E stained sections and calculated by using the following formula: (∑ total plaque length/∑ total vessel length) × 100. Immunohistochemical staining for anti-VWF (von Willebrand factor; PC054; Binding Site, San Diego, CA, USA) and Ter-119 (anti–Ter-119, 550565; BD Biosciences, Franklin Lakes, NJ, USA) were performed to detect intraplaque endothelial cells (microvessels) and erythrocytes (bleeding), respectively. All images were acquired with Universal Grab 6.1 software using an Olympus BX40 microscope and were quantified with ImageJ software (NIH, Bethesda, MA, USA).

### 4.7. Statistical Analyses

Normally distributed data are expressed as mean ± SEM and non-normally distributed variables are represented as median [min-max]. Statistical analyses were performed using SPSS software (version 28, SPSS Inc., Chicago, IL, USA). Statistical tests are specified in the figure and table legends. If a two-way ANOVA or a three-way mixed ANOVA was used, a significant main effect was further analysed by performing a simple main effect analysis, including a Bonferroni correction for multiple comparisons. Mice that died during the experiment were excluded from body and organ weight analyses. Histological data of the proximal ascending aorta, coronary arteries and the heart only include mice that survived until 20 weeks of WD in order to avoid disease stage related variations. Differences were considered significant at *p* < 0.05.

## Figures and Tables

**Table 1:** Main characteristics of control and ASA-treated *ApoE^−/−^* and *ApoE^−/−^Fbn1^C1039G+/−^* mice.

	*ApoE^−/−^*		*ApoE^−/−^Fbn1^C1039G+/−^*		Main Effect *p* Values		
	Control	ASA	Control	ASA	G	T	I
Mortality (%) ^1^	0 (0/15)	0 (0/14)	52 (11/21)	58 (11/19)	1 × 10^−6^	0.805	/
Cholesterol (mg/dL) ^2^	440 ± 26	414 ± 24	488 ± 40	423 ± 25	0.359	0.146	0.537
Body weight (g) ^2^	23.3 ± 0.7	22.5 ± 0.7	20.5 ± 0.7 ^§^	19.5 ± 0.9	0.001	0.244	0.870
Heart weight/BW (%) ^2^	0.50 ± 0.02	0.49 ± 0.02	1.16 ± 0.12 ^§§§^	0.76 ± 0.05 ***	3 × 10^−13^	0.002	0.004
Lung weight/BW (%) ^2^	0.69 ± 0.03	0.68 ± 0.02	0.99 ± 0.09 ^§§§^	0.87 ± 0.04	4 × 10^−6^	0.280	0.431
Spleen weight/BW (%) ^2^	0.48 ± 0.03	0.40 ± 0.02	0.81 ± 0.10 ^§§§^	0.58 ± 0.07 *	2 × 10^−5^	0.004	0.341

^1^ Pearson Chi-Square test. ^2^ 2-way ANOVA: ^§^ *p* < 0.05, ^§§§^ *p* < 0.001 vs. *ApoE^−/−^* control and * *p* < 0.05, *** *p* < 0.001 vs. *ApoE^−/−^Fbn1^C1039G+/−^* control. G = genotype; T = treatment; I = interaction; BW = body weight.

**Table 2:** Blood immune cell profile.

	*ApoE^−/−^*		*ApoE^−/−^Fbn1^C1039G+/−^*		Main Effect *p* Values		
	Control	ASA	Control	ASA	G	T	I
Dendritic cells (cells/µL)	130 ± 26	99 ± 25	78 ± 23	76 ± 18	0.161	0.537	0.587
Ly-6C^hi^ monocytes (cells/µL)	397 ± 48	391 ± 50	686 ± 111 ^§§^	521 ± 54	0.004	0.219	0.254
Ly-6C^lo^ monocytes (cells/µL)	451 ± 55	440 ± 78	499 ± 84	536 ± 74	0.341	0.859	0.744
T cells (cells/µL)	1095 ± 152	1129 ± 272	879 ± 160	1325 ± 243	0.965	0.281	0.354
B cells (cells/µL)	2044 ± 240	2480 ± 488	2157 ± 562	3201 ± 635	0.379	0.122	0.520
NK cells (cells/µL)	463 ± 48	426 ± 67	539 ± 73	466 ± 101	0.414	0.440	0.796
NKT cells (cells/µL)	63 ± 12	81 ± 24	64 ± 12	68 ± 10	0.724	0.516	0.694

Data are represented as the number of cells per µL blood. 2-way ANOVA: ^§§^ *p* < 0.01 vs. *ApoE^−/−^* control. G = genotype; T = treatment; I = interaction.

**Table 3:** Atherosclerotic plaque and MI characteristics.

		*ApoE^−/−^*		*ApoE^−/−^Fbn1^C1039G+/−^*		Main Effect *p* Values		
		Control	ASA	Control	ASA	G	T	I
Proximal ascending aorta	Plaque size (10^3^ µm^2^) ^1^	522 ± 47	467 ± 28	1255 ± 135 ^§§§^	1229 ± 153	1 × 10^−8^	0.578	0.818
	Stenosis (%) ^1^	46.6 ± 3.2	44.4 ± 1.9	60.3 ± 1.5 ^§§^	58.0 ± 3.4	4 × 10^−5^	0.444	0.979
	IEL length (µm) ^1^	3740 ± 93	3558 ± 57	4776 ± 259 ^§§^	5063 ± 288	3 × 10^−6^	0.988	0.347
	Necrotic core (%) ^1^	15.1 ± 2.3	11.8 ± 2.7	18.2 ± 2.1	19.6 ± 3.7	0.082	0.765	0.452
	SMCs (%) ^1^	5.6 ± 0.8	6.4 ± 1.0	4.9 ± 0.6	4.9 ± 0.6	0.130	0.575	0.558
	Cap thickness (µm) ^1^	12.1 ± 3.1	9.9 ± 1.8	9.1 ± 1.0	12.5 ± 1.4	0.907	0.713	0.125
	Total collagen (%) ^1^	67.9 ± 2.2	68.7 ± 1.8	64.5 ± 2.1	61.2 ± 2.2	0.020	0.588	0.370
	Glycosaminoglycans (%) ^1^	51.2 ± 2.0	56.0 ± 2.4	50.4 ± 2.9	53.3 ± 2.0	0.501	0.148	0.717
	p-SMAD2/3 positive nuclei (%) ^1^	67.5 ± 5.6	68.1 ± 1.4	67.8 ± 3.5	58.5 ± 3.3	0.443	0.796	0.588
	Leukocytes (%) ^1^	6.0 ± 0.6	7.1 ± 0.6	6.7 ± 0.5	8.1 ± 0.8	0.204	0.080	0.819
	Macrophages (%) ^1^	4.1 ± 0.6	3.8 ± 0.4	3.5 ± 0.4	2.9 ± 0.4	0.114	0.260	0.732
Carotid artery	Plaque formation index (%) ^1^	55 ± 6	61 ± 7	83 ± 3 ^§§^	79 ± 4	1 × 10^−4^	0.844	0.364
	IP microvessel occurrence (%) ^2^	8	25	36	46	0.060	0.311	/
	IP bleeding occurrence (%) ^2^	8	25	29	38	0.173	0.296	/
Coronary arteries—MI	Plaque occurrence (%) ^2^	47	43	44	47	0.979	0.979	/
	Plaque size (µm^2^) ^1^	9515 ± 4706	7616 ± 2417	4043 ± 516	5518 ± 2378	0.222	0.944	0.581
	Stenosis (%) ^1^	50 ± 17	69 ± 13	47 ± 7	37 ± 8	0.158	0.699	0.237
	MI occurrence (%) ^2^	13	14	38	40	0.029	0.876	/

^1^ 2-way ANOVA: ^§§^ *p* < 0.01, ^§§§^ *p* < 0.001 vs. *ApoE^−/−^* control. ^2^ Pearson Chi-Square test. G = genotype; T = treatment; I = interaction; IEL = internal elastic lamina; SMCs = smooth muscle cells; IP = intraplaque; MI = myocardial infarction.

## Data Availability

The data presented in this study are available on request from the corresponding author.

## Supplementary Materials

The following supporting information can be downloaded at: https://www.mdpi.com/article/10.3390/ijms23010404/s1.
