# Supplementary material for: Acetylsalicylic Acid Reduces Passive Aortic Wall Stiffness and Cardiovascular Remodelling in a Mouse Model of Advanced Atherosclerosis
Source: Int J Mol Sci. 2021 Dec 30;23(1):404. doi: 10.3390/ijms23010404 (PMC8745264; doi:10.3390/ijms23010404)
Supplement: Supplementary file 1 [file ijms-23-00404-s001.zip › ijms-1300333-supplementary.pdf]

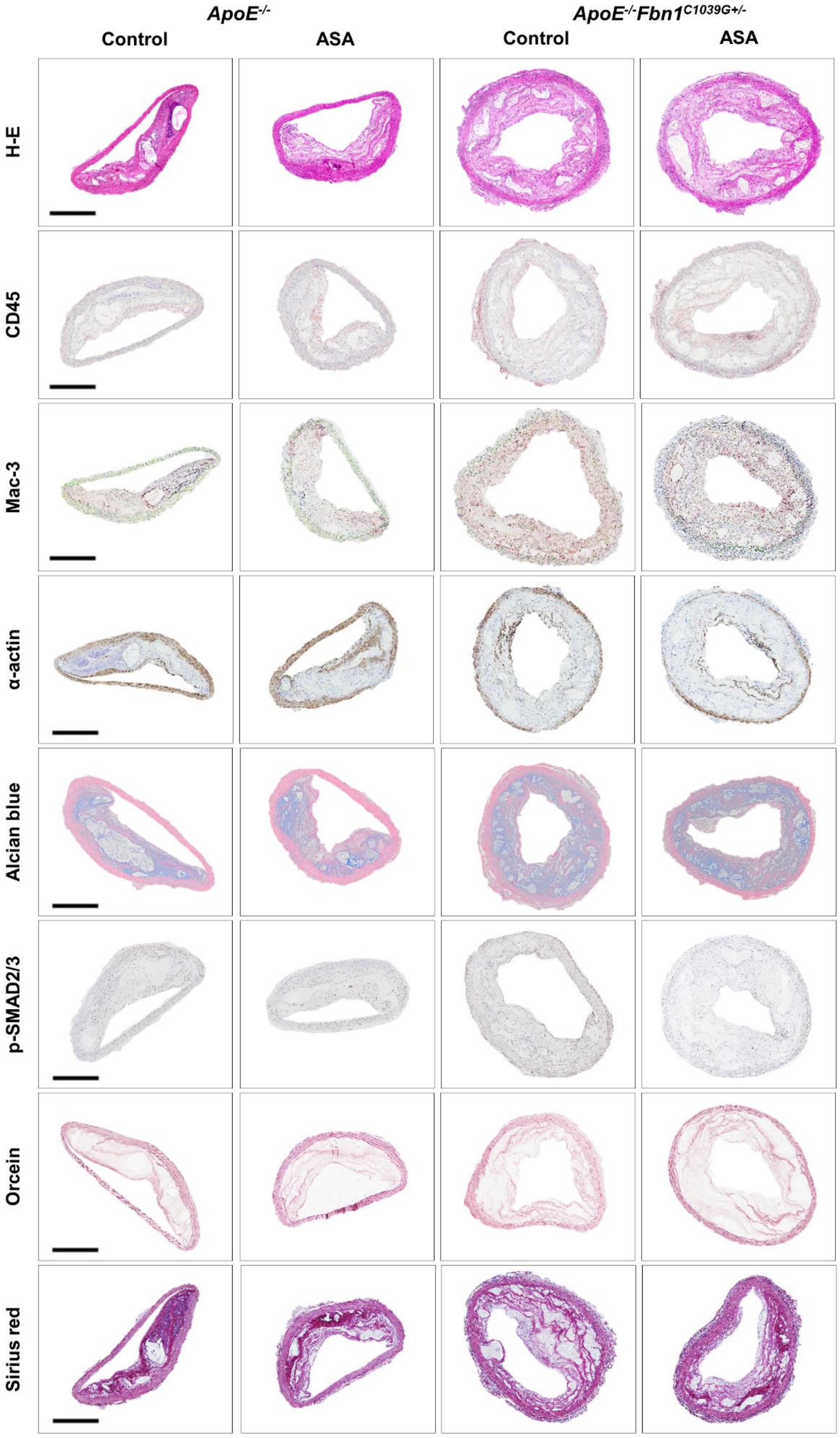

Figure S1. Representative images of the different stainings performed on the proximal ascending aorta of *ApoE*$^{-/-}$ and *ApoE*$^{-/-}$*Fbn1*$^{C1039G+/-}$ mice fed a Western diet in combination with plain or ASA-supplemented drinking water. Scale bar = 500 µm.
